# The association between healthy beverage index and sarcopenia in Iranian older adults: a case-control study

**DOI:** 10.1186/s12877-024-04790-z

**Published:** 2024-03-11

**Authors:** Marzieh Mahmoodi, Zainab Shateri, Mehran Nouri, Mohebat Vali, Nasrin Nasimi, Zahra Sohrabi, Mohammad Hossein Dabbaghmanesh, Maede Makhtoomi

**Affiliations:** 1 Department of Clinical Nutrition, School of Nutrition and Food Sciences, Shiraz University of Medical Sciences, Shiraz, Iran; 2 Student Research Committee, Shiraz University of Medical Sciences, Shiraz, Iran; 3 Department of Nutrition and Biochemistry, School of Medicine, Ilam University of Medical Sciences, Ilam, Iran; 4 Health Research Institute, Babol University of Medical Sciences, Babol, Iran; 5 Department of Community Nutrition, School of Nutrition and Food Sciences, Shiraz University of Medical Sciences, Shiraz, Iran; 6 Nutrition Research Center, School of Nutrition and Food Sciences, Shiraz University of Medical Sciences, Shiraz, Iran; 7 Endocrinology and Metabolism Research Center, Shiraz University of Medical Sciences, Shiraz, Iran; 8 Health Policy Research Center, Institute of Health, Shiraz University of Medical Sciences, Shiraz, Iran

**Keywords:** Healthy beverage index, Sarcopenia, Older adults, Iranian

## Abstract

**Background:**

Sarcopenia is a progressive disease with age-related loss of skeletal muscle mass, strength, and function. No study has investigated the association between healthy beverage index (HBI) and sarcopenia in older adults. Therefore, the present study aimed to investigate the association between HBI and sarcopenia in Iranian older adults.

**Methods:**

In the present case-control study, 80 sarcopenic and 80 non-sarcopenic participants matched in sex were included. Body composition was measured using bioelectrical impedance analysis. Handgrip strength (HGS), skeletal muscle mass index (SMI), and gait speed were utilized to confirm sarcopenia. Also, a food frequency questionnaire was used to evaluate food intake. HBI score was calculated based on ten sub-components of the total beverages. Moreover, logistic regression was applied to assess the association between HBI and sarcopenia.

**Results:**

In the crude model, we observed no significant association between HBI and the odds of sarcopenia. Still, after adjusting the confounders, the odds of developing sarcopenia decreased significantly in the second and last tertiles (T) (T_2_– odds ratio (OR) = 0.04, 95% confidence interval (CI): 0.01–0.25 and T_3_– OR = 0.10, 95% CI: 0.01–0.60).

**Conclusions:**

Our findings indicated that HBI is inversely related to the chance of sarcopenia. Therefore, to reduce the odds of sarcopenia, it is recommended to consume healthy drinks such as fruit juices and milk.

## Introduction

Sarcopenia is a progressive and generalized skeletal muscle disorder with age-related loss of skeletal muscle mass, strength, and function [1, 2]. Therefore, this condition is related to an increased likelihood of adverse outcomes, including falls, fractures, and more mortality and morbidity in older people [1]. Also, sarcopenia can lead to decreased mobility, physical inactivity, decreased walking speed, and reduced endurance [3]. The estimated prevalence of sarcopenia in Western societies and Asian countries has been reported as 1–29% and 2–46%, respectively [4]. The findings of a study estimated the prevalence of sarcopenia in Iran between 16.5% and 32.5% [5].

Nutrition and physical activity are essential in managing and preventing sarcopenia [5]. There is considerable evidence that nutrition plays an essential role in the strength, performance, and muscle mass of the elderly [6]. A healthy diet, such as a lower intake of added sugar and a higher intake of whole fruit, is related to muscle strength [7]. It has also been reported that sugar-sweetened beverages (SSBs) are associated with the loss of muscle mass [8]. Beverage patterns are related to health and dietary patterns [9]. The healthy beverage index (HBI) can be applied to evaluate the total quality of beverages and reveal whether changing beverage patterns are associated with further health [10]. This index can be used to evaluate the quality of people’s nutrition and drink consumption to help individuals choose healthy drinks [11]. Also, the HBI can be used to examine the synergistic effects of different beverages rather than the effect of a single beverage on health-related outcomes [12]. This index includes fluid intake, total beverage energy, and eight beverage categories [13].

Studies have shown SSBs could harm muscle mass [8, 14]. In contrast, the positive effect of caffeine on handgrip strength (HGS) and improved muscle function and physical performance has been shown [15, 16]. It has also been demonstrated that beverages like milk, green tea, and coffee can positively increase lean body mass [17–19]. Moreover, it has been shown that consumption of normal/high-fat products is associated with greater muscle mass and lean body mass in Japanese women aged 40–60 years [20]. In addition, a study found a positive relationship between coffee consumption and muscle mass in older and middle-aged Japanese people [21]. However, another study found no relationship between coffee consumption and muscle mass [22].

Considering that the number of elderly people is increasing in the new century, it is crucial to find approaches to prevent sarcopenia to avoid the epidemic of disability in the future [23]. To our knowledge, no study has investigated the association between HBI and sarcopenia in older adults. Therefore, the present study aimed to investigate the association between HBI and sarcopenia in Iranian older adults. The results of the present study can help us understand the relationship between the consumed beverages and the odds of sarcopenia.

## Methods

### Study population

This case-control study is a subset of a previous population-based cross-sectional study conducted on community-dwelling older adults referred to healthcare centers in Shiraz, Iran, from August 2017 to February 2018 [24]. The diagnosis of sarcopenia was based on the prevalence (existing cases at the beginning of the study) and the participants were selected from among 501 older adults. Of the 501 eligible participants who entered the cross-sectional study, 104 sarcopenic and 397 non-sarcopenic patients were diagnosed by physicians [24]. In the present case-control study, according to the inclusion and exclusion criteria, the case group (*n* = 80) was selected by the available sampling method from among the identified sarcopenic participants who were referred to the healthcare centers of Shiraz in the previous cross-sectional study [24]. The control group (*n* = 80) was also selected by matching based on gender and using the available sampling method from the older people without sarcopenia who were referred to Shiraz’s health centers in the previous cross-sectional study [24].

In the current study, the inclusion criteria were age over 65 years, independently living, physically active, and having no history of severe cardiac, pulmonary, or musculoskeletal diseases, severe neurological disorders, stroke, malignancies, or any acute organ failure, and cognitive problems. The case and control participants with incomplete questionnaires or unwillingness to participate in the research were also excluded from the study.

Basic demographic information such as sex, age, education, and smoking status were gathered using a general information checklist. Also, income classification was done according to Iran’s economic situation (based on Iranian rials (IRR)) [2]. Body mass index (BMI) was determined by dividing the body weight obtained from a digital scale with an accuracy of 100 g by the square of height. Physical activity was evaluated and reported in this study using the international physical activity questionnaire (IPAQ), which was previously validated [25].

All participants completed an informed consent form. This study was approved by the Ethics Committee of Shiraz University of Medical Sciences and performed in line with the principles of the Declaration of Helsinki (IR.SUMS.SCHEANUT.REC.1402.121).

### Sarcopenia diagnosis

According to the Asian Working Group for Sarcopenia (AWGS) guidelines, sarcopenia was defined as a reduction in skeletal muscle mass and muscle function (including low muscle strength and/or low physical performance) [26]. Skeletal lean mass and segmental lean mass of arms, trunk, and legs, as well as other components of body composition, were measured using bioelectrical impedance analysis (BIA) InBody S10 analyzer (BioSpace Co., Ltd., South Korea). Skeletal muscle mass index (SMI) [27] was defined as appendicular skeletal muscle mass [28] (as the sum of segmental muscle mass values of the legs and arms) divided by the squared height (meters). SMI < 7 kg/m^2^ for men and < 5.7 kg/m^2^ for women were considered low skeletal muscle mass [24, 26, 29].

Muscle strength was defined by HGS with a hydraulic hand dynamometer (model MSD, Sihan, Korea). The participants squeezed a hand dynamometer in both hands three times with 15-second pauses in a seated position. The maximum value was used for further analyses. HGS values < 18 kg for women and < 26 kg for men were determined as low muscle strength [24, 26, 29].

Physical performance was also evaluated by measuring the usual walking speed for 4 m (gait speed). Gait speed values < 0.8 m/s were considered as a low physical performance [24, 26, 29].

### Dietary assessment and food grouping

A semi-quantitative food frequency questionnaire (FFQ) including 168 foods with standard and common serving sizes used by Iranians was applied to evaluate participants’ dietary intake. According to previous studies, this questionnaire has good reproducibility and validity in the Iranian population [30]. It was completed by a trained dietitian using a face-to-face interview. A valid food album and standard measurement tools were used to assist participants in estimating dietary intake. Participants reported their daily, weekly, monthly, or yearly intake of foods or food items during the past year, and all data were converted to grams. Finally, the results obtained from multiplying the frequency of each food’s daily consumption by the portion size were reported as the consumption of each food. The content of energy, micronutrients, and macronutrients in foods were also calculated using the Iranian-modified version of Nutritionist software.

The HBI was calculated based on the following ten subcomponents of total beverages, including water (0–15 points), low-fat milk (< 1.5% fat or fat-free) (0–5 points), full-fat milk (˃1.5% fat) (0–5 points), tea and unsweetened coffee (0–5 points), diet drinks (artificially sweetened and calorie-free beverages) (0–5 points), natural fruit juice (0–5 points), alcohol (beer, liquor, and wine) (0–5 points), SSBs(soda and sweetened coffee) (0–15 points), total beverage energy (0–20 points) and met fluid requirement (0–20 points). Also, Duffey and Davy’s method [12] suggested that a higher score of total HBI is associated with better adherence to healthier HBI pattern. The final score of HBI was 90 because of non-reporting the alcohol content and diet drinks [31].

### Statistical analysis

In the present study, statistical analysis was performed using SPSS (version 24). A statistical significance level was considered as < 0.05. The Kolmogorov-Smirnov test was used to assess the normality of the data. The data were reported as mean ± standard deviation (SD) or median and interquartile range (IQR) and frequency or percentage for continuous and categorical variables, respectively. The comparison of the study population’s baseline continuous and categorical variables between the case and the control groups was analyzed using the independent samples T-test (for data with normal distribution) or Mann-Whitney U-test (for skewed data), and chi-square test, respectively. Also, multiple logistic regression models were used to assess the relationship between HBI and sarcopenia. Furthermore, graphs were depicted by R software.

## Results

Baseline characteristics of the study population are shown in Table 1. According to the table, the median age significantly differed between both groups (*P* = 0.001). Also, energy, fiber, water and tea consumption, weight, height, BMI, muscle strength, SMI, and gait speed were different between the case and control groups (P<0.001 for all except gait speed).

**Table 1:** The basic characteristics of the study participants

Variables	Case (*n* = 80)	Control (*n* = 80)	*P*-value
Age (year) ^1^	70.0 (8.0)	68.0 (6.0)	**0.001**
Total HBI score ^1^	80.0 (4.0)	80.0 (4.0)	0.104
Energy (kcal/day) ^2^	1329.3 ± 472.9	1861.9 ± 450.4	**<0.001**
Fiber (gr/day) ^2^	26.1 ± 12.5	36.1 ± 11.2	**<0.001**
Water (cc/day) ^1^	720.0 (700.0)	1000.0 (915.0)	**<0.001**
Tea (cc/day) ^1^	500.0 (250.0)	1000.0 (500.0)	**<0.001**
Low-fat milk (cc/day) ^2^	54.8 ± 10.2	69.9 ± 11.3	0.324
High-fat milk (cc/day) ^2^	5.7 ± 4.0	1.2 ± 1.2	0.288
Fruit juice (cc/day) ^2^	2.2 ± 1.6	0.0 ± 0.0	0.174
Sugar-sweetened beverages (cc/day) ^2^	24.7 ± 9.5	5.7 ± 1.6	0.052
Weight (kg) ^2^	59.6 ± 9.2	78.1 ± 9.1	**<0.001**
Height (cm) ^2^	157.2 ± 9.8	163.6 ± 8.9	**<0.001**
BMI (kg/m^2^) ^2^	24.5 ± 4.1	29.2 ± 3.8	**<0.001**
Physical activity (MET-h/week) ^1^	429.0 (1020.7)	462.0 (1386.0)	0.650
Sex, % ^3^			1.000
Male	55.0	55.0	
Female	45.0	44.0	
Education, % ^3^			0.053
Illiterate	31.3	13.8	
Primary education	33.7	47.5	
Secondary education	22.5	22.5	
Higher education	12.5	16.3	
Income in month, % ^3^			0.127
Less than 30 million IRR	38.8	36.3	
30–60 million IRR	47.5	37.5	
More than 60 million IRR	13.7	26.2	
Smoking, % ^3^			0.718
Yes	23.7	27.5	
No	76.3	72.5	
Muscle strength (kg) ^1^	16.0 (10.7)	50.8 (24.2)	**<0.001**
SMI (kg/m^2^) ^1^	6.1 (1.4)	7.9 (0.9)	**<0.001**
Gait speed (m/second) ^2^	0.70 ± 0.10	1.00 ± 0.95	**0.007**

BMI: body mass index, HBI: healthy beverage index, IRR: Iranian rial, SMI: skeletal muscle mass index, MET: metabolic equivalent of task

Values are median (IQR), mean ± SD, or percentage.

*P*-value less than 0.05 was considered significant.

^1^ Mann-Whitney U test has been used.

^2^ Independent samples T-test has been used.

^3^ Chi-square test has been used.

The nutrient intakes of the study population in various tertiles of HBI are shown in Table 2. Participants in the last tertile of the HBI score had significantly higher intakes of polyunsaturated fatty acids (PUFAs) and monounsaturated fatty acids (MUFAs) than those in thefirst tertile (*P* = 0.031 and *P* = 0.049, respectively), but carbohydrate intake was significantly lower in the last tertile (*P* = 0.032).

**Table 2:** The nutrient intakes based on HBI tertile

Variables	T1 (*n* = 60)	T2 (*n* = 62)	T3 (*n* = 38)	*P*-value^1^
Carbohydrate (% energy)	65.57 (9.60)	61.83 (11.72)	63.50 (9.95)	**0.032**
Protein (% energy)	13.47 (2.82)	13.51 (3.13)	14.15 (2.25)	0.334
SFA (% energy)	7.45 (3.44)	7.96 (4.12)	7.65 (3.10)	0.552
MUFA (% energy)	8.06 (2.27)	9.29 (3.50)	8.46 (3.65)	**0.049**
PUFA (% energy)	5.23 (2.03)	5.90 (1.79)	5.30 (2.65)	**0.031**

HBI: healthy beverage index, T: tertile, SFA: saturated fatty acids, MUFA: monounsaturated fatty acids, PUFA: polyunsaturated fatty acids

Values are median (IQR).

*P*-value less than 0.05 was considered significant.

^1^ Kruskal-Wallis U-test has been used.

Crude and multivariable-adjusted odds ratio (OR) and 95% confidence intervals (CIs) for HBI score with the odds of sarcopenia are presented in Table 3. In the crude model, no significant relationship was observed between HBI and the odds of sarcopenia. Still, after adjusting for age, BMI, smoking history, education level, income, physical activity, energy, protein and saturated fatty acid (SFA) intake (energy%), the odds of developing sarcopenia decreased significantly in the second and last tertiles (T) (T_2_– OR = 0.04, 95% CI: 0.01–0.25 and T_3_– OR = 0.10, 95% CI: 0.01–0.60).

**Table 3:** Association between healthy beverage index and sarcopenia

Tertiles of Indices	Case/Control	Model 1		Model 2		Model 3	
		OR	95% CI	OR	95% CI	OR	95% CI
**Healthy beverage index**							
T_1_ (≤ 77)	33/27	1.00	Ref.	1.00	Ref.	1.00	Ref.
T_2_ (78–80)	31/31	0.81	0.40–1.66	0.77	0.32–1.87	**0.04**	**0.01–0.25**
T_3_ (≥ 81)	16/22	0.59	0.26–1.35	0.72	0.26–1.99	**0.10**	**0.01–0.60**
P_trend_		0.219		0.588		**0.020**	

Model 1: crude model

Model 2: adjusted for age, BMI, and income

Model 3: adjusted for age, BMI, smoking history, education level, income, physical activity, energy, protein, and SFA intake (energy%)

-Obtained from logistic regression

-These values are odds ratio (95% CIs).

-Significant values are shown in bold.

## Discussion

The current study’s findings indicated a significant negative association between HBI and sarcopenia in older adults. The chance of sarcopenia was decreased by 90% with a higher HBI score.

In most elderly patients, the onset of sarcopenia is multifactorial [32], including weight loss, an increase of pro-inflammatory cytokines, loss of anabolic hormones, age-related mitochondrial dysfunction, reduction of physical activity, and loss of motor neuron end plates [33]. Sarcopenia caused by these factors can negatively affect the general health of the elderly. Sarcopenia is related to functional decline and poor physical performance, which can cause an increase in hospitalization, an increase in co-morbidities, and disability [34].

Based on the current study’s findings, the odds of sarcopenia decreased with increasing HBI. A study by Guo et al. showed that caffeinated coffee consumption in elderly mice prevents the decline of muscle strength and muscle weight, can reduce pro-inflammatory mediators, and has beneficial effects on reducing the risk of age-related sarcopenia [35]. Also, a study by Kim et al. indicated a 31% reduction in the risk of sarcopenia with one cup of coffee per day in men [36]. Coffee contains chemical compounds with antioxidant and anti-inflammatory properties that can cause autophagy and have beneficial effects on reducing the risk of sarcopenia [36]. Catechin and polyphenols in green tea cause its antioxidant properties, which may be associated with a reduced risk of sarcopenia [37, 38].

Studies have also been conducted on the effect of milk consumption on sarcopenia. A cross-sectional study in Korea has shown that milk consumption significantly reduces performance disability in men [39]. Also, another cross-sectional study demonstrated that milk consumption was associated with increased skeletal muscle mass, fat-free mass, and HGS in elderly women [40]. Also, a systematic review study demonstrated that the consumption of low-fat milk can be useful in reducing the risk of sarcopenia in the elderly by improving skeletal muscle mass due to its protein and nutrients [41]. Milk contains many bioactive components and nutrients that may benefit muscles; for example, it contains proteins useful for muscle synthesis and has muscle-protective properties [42]. Also, milk contains some antioxidant elements, such as β-lactoglobulin, which positively impact sarcopenia [42].

Fruit juice consumption is another component of the HBI, which has been shown to reduce sarcopenia [43, 44]. Oxidative stress is an important factor that causes metabolic disorders and changes in muscle function [45]. Oxidative stress can play a role in the pathophysiology of sarcopenia and can increase its risk [46]. The beneficial effects of fruits in reducing oxidative stress have been demonstrated in previous studies [47]. Fruits owe their antioxidant properties to vitamins C and E, phenolic compounds, and carotenoids, which destroy free radicals and prevent damage to deoxyribonucleic acid (DNA) and cellular structures [48–50]. In addition, fruit juices act like a buffer with their alkaline properties, reduce catabolism and proteolysis of amino acids, and increase muscle mass [51]. We can also mention vitamin C’s role in synthesizing collagen and carnitine in skeletal muscle, which can be useful in reducing the risk of sarcopenia [52–54].

Regarding the consumption of SSBs, studies illustrate that limiting their consumption is related to the increase in the expression of mitophagy-related proteins in the quadriceps muscles [55]. A study by Bragança et al. also indicated that daily consumption of SSBs was associated with a decrease in muscle mass index [14]. Moreover, a study by Hao et al. showed that SSB consumption is related to a decline in muscle mass by 0.12 kg/m^2^ in adolescents [8]. Studies reveal that muscle fat increases with increased SSB consumption [56, 57]. An increase in fat in muscle cells could simultaneously increase lipolysis and autophagy in muscle cells [58]. Impairment of autophagy reduces myogenesis and causes a decrease in muscle mass [59]. Also, the role of a diet with higher sugar has been shown to reduce the function of mitochondria and muscle cells [60]. Also, the consumption of SSBs is associated with the loss of muscle mass and, thus, the risk of sarcopenia through their effects on impaired glucose, lipid metabolism, reduced protein synthesis, and decreased efficient muscle contraction [61–64].

In general, HBI components seem to reduce the risk of sarcopenia by decreasing oxidative stress, anti-inflammatory effects, having bioactive nutrients, and increasing protein synthesis in muscles.

This study had some limitations and strengths. Since the nature of the study is case-control, we cannot directly determine causation, but the association between exposure (HBI) and outcome (sarcopenia) was assessed. Also, there may be other confounding variables that were not considered in the present study, such as polypharmacy, which is common in older adults. In addition, completing the FFQ relies on people’s memory, and there may be errors in dietary assessment. In terms of the strength of the current study, it can be mentioned that this study is the first case-control study that examined the association between HBI and the odds of sarcopenia in older adults.

In this way, in this type of study, although causation cannot be determined directly; instead, researchers can identify associations and calculate measures such as odds ratios to estimate the strength of the association between exposure and outcome.

## Conclusions

Our findings indicated that HBI had an inverse relationship with sarcopenia risk. These findings showed the importance of beverages as an important dietary factor contributing to health. It can also advise people to consume beverages such as low-fat milk and fruit juices and avoid certain beverages such as SSBs to experience healthy aging. However, more studies are needed to confirm these results and better elucidate the relationship between nutrient and beverage consumption and sarcopenia variables.

## Data Availability

Data are available through a reasonable request from the corresponding author.
